# A Disputed Hegemony: Negotiating Neurosurgical Patient Care in the Netherlands, 1930–1952

**DOI:** 10.1093/jhmas/jrae014

**Published:** 2024-08-06

**Authors:** Bart Lutters

**Affiliations:** University Medical Center Utrecht, Utrecht, The Netherlands

**Keywords:** professional identity, jurisdiction, professionalization, neurosurgery, neurology, the Netherlands

## Abstract

The emergence of the neurosurgical patient as a novel clinical entity in the Netherlands was marked by a lingering conflict between neurologists and neurosurgeons, in which both types of specialists sought to assume the clinical and institutional leadership of neurosurgical patient care. In the 1920s and 1930s, neurologists had facilitated the establishment of the first generation of neurosurgeons in the country, and in the process, had managed to clinically and institutionally subordinate neurosurgery to neurology. As the demand for neurosurgical patient care grew, the neurosurgeons began to challenge this hegemonic relationship. The neurologists, however, were unwilling to give up their control, fearing that they would be bypassed in the diagnosis of patients eligible to neurosurgery. These conflicting aims and interests resulted in an intricate demarcation battle, in which the boundary work between neurologists and neurosurgeons was directly played out at the local workplace and at the meetings of the Study Club for Neuro-Surgery, and indirectly at various other sites of contestation, such as medical journals and academic lecture halls, as both parties sought to rally external stakeholders to their cause. During these negotiations, local, national, and international forces increasingly intertwined to shape the particular organization of Dutch neurosurgery in the middle of the twentieth century. By analyzing this multilayered demarcation process, this article draws attention to the complexity of medical boundary work, and to the way in which, despite pervasive international influences, specialist practice was ultimately negotiated at the local and national levels.

In November 1950, the Dutch neurosurgeon Arnold de Vet (1904-2001) wrote a letter to the other neurosurgeons in the country about an invitation they had all received from their neurological colleagues to form a neurosurgical subdivision of the Netherlands Society for Psychiatry and Neurology (*Nederlandsche Vereeniging voor Psychiatrie en Neurologie*). In his letter, De Vet encouraged his fellow neurosurgeons to decline the invitation, as “a situation will arise similar to the wrong state of affairs in our clinics, where the neurosurgery ward […] is under supervision of the neurologist, who makes the decisions and has to represent us for the authorities, making us dependent on whatever he deems necessary and acceptable, so that we can never directly stand up for our own interests.”^1^

De Vet’s comments were representative of the lingering conflict between neurosurgeons and neurologists about the organization of neurosurgical patient care in the Netherlands in the decades around World War II. During this period, specialist medicine evolved from a predominantly local into a national phenomenon, as the rapid increase in specialists posed central issues related to specialist training, certification, and jurisdiction.^2^ The boundary work between neurologists and neurosurgeons that ensued upon the arrival of neurosurgery on the crowding medical marketplace was not unique to the Dutch context.^3^ In the interwar period, American neurosurgeons and neurologists were involved in similar disputes, in which both types of specialists mobilized their professional identity to promote their particular interests, thereby evoking specific arguments and metaphors with broad cultural resonance, such as claims to therapeutic superiority, particular readings of history, and appeals to fundamental differences in character between physicians and surgeons.^4^ Neurological and neurosurgical specialist societies were important sites for crafting and performing these identities by facilitating the exchange of knowledge, skills, and norms of conduct among a select group of peers.^5^

In the Netherlands, specialist medical practice emerged in the major cities at the close of nineteenth century, primarily in outpatient settings.^6^ During the first decades of the twentieth century, the legitimacy, jurisdiction, and reimbursement of specialists remained contested by general physicians, who dominated the Dutch Medical Association (*Nederlandsche Maatschappij tot bevordering der Geneeskunst*, NMG) and were generally dismissive of specialist interests. By the 1930s, the growing demand for specialized medicine, the specialists’ turn towards the hospital, and the foundation of specialist societies and collective specialist associations gradually strengthened the position of the Dutch specialists, as exemplified by the installment of a formal Specialist Registry by the NMG in 1932. Still, the diverging interests of individual specialties continued to undermine a mutual sense of solidarity, and made the emergence of new specialties such as neurosurgery into a controversial phenomenon amongst the specialists involved. Since the late nineteenth century, nervous system surgery in the Netherlands had been characterized by a division of labor between surgeons and neurologists, who had only reluctantly come to acknowledge the need for a new specialist dedicated to this type of surgery.^7^ By the 1920s, however, the Dutch neurologists began to recognize the considerable therapeutic promise of the neurosurgeon, and set out to establish neurosurgery wards within their neurological or psychiatric-neurological clinics, thereby seeking to physically and intellectually incorporate neurosurgery as part of neurology to infuse their field with therapeutic potency. The subordination of neurosurgery to neurology also found expression in the Study Club for Neuro-Surgery (*Studieclub voor Neuro-chirurgie*), the first professional club dedicated to neurosurgery, which was chaired by a neurologist and had an equal number of neurosurgeons and neurologists as members. This particular organization of Dutch neurosurgery set the stage for a fierce and multifaceted demarcation battle, in which neurosurgeons increasingly strove for clinical and institutional independence, while the neurologists were unwilling to give up their control, fearing that they would be bypassed in the diagnosis and localization of patients eligible to neurosurgery.

These conflicting interests first gave rise to contestation at the local workplace, where neurosurgeons sought to persuade hospital directors and university curators of their need for more clinical autonomy. But the boundary work soon spread to external sites of contestation, such as medical journals and academic lecture halls, as neurosurgeons and neurologists both acknowledged the need to rally external stakeholders in order to strengthen their jurisdictional claim over the neurosurgical patient. In doing so, the neurosurgeons drew on stories similar to those employed by their American colleagues, but some were also unique to the Dutch context, such as frequent comparisons with the state of neurosurgery abroad, denouncing the division of labor between surgeons and neurologists in the past, and the idolization of the American pioneer of neurosurgery Harvey Cushing (1869-1939).^8^ In addition, the rise of invasive diagnostic techniques enabled the Dutch neurosurgeons to redefine the challenge of diagnosing and localizing nervous system disorders in their own terms, effectively drawing the diagnostic process into their own jurisdictional domain.

Besides these ongoing lobbying efforts in the public arena, the neurosurgeons and neurologists also privately faced one another at the meetings of the Study Club, where the particular talents and temperaments of neurological and neurosurgical members were directly played out and increasingly clashed as professional issues began to surface. As the mutual tensions grew and the negotiations in the Study Club threatened to reach a stalemate, both parties intensified their quest to convince local and national authorities of their particular views on the proper organization of neurosurgical patient care in the country. This multilayered demarcation process was importantly shaped by the divergent views and interests of the different external stakeholders, the strong competitive forces between Dutch hospitals and universities, and pervasive international developments, such as the expansion of neurosurgical indications and the devasting impact of the war.^9^ In the negotiation of neurosurgical patient care in the Netherlands, then, the distinctions among local, national and international forces and actors increasingly blurred, effectively dissolving the strict dichotomy between the micro and the macro level of medical boundary work.

In this article, I seek to expose this intricate demarcation process. In doing so, I draw on Andrew Abbott’s influential theory of professional development, as its focus on interprofessional competition, the actual work professions compete for, and the quest for external validation are particularly useful to my analysis.^10^ According to Abbott, the link between a profession and its work, which he referred to as “jurisdiction,” is constantly reconfigured within an interdependent system of competing professional forces. Within this system, the successful profession manages to gain control over certain tasks because its members succeed in convincing the outside world of their ability to perform those tasks better than others, particularly by defining existing problems in new ways that draws them firmly into the profession’s own domain, a professional strategy he called “abstraction.”^11^ The Netherlands provides a potent case study for examining the complex set of local, national, and international forces that shaped the organization of neurosurgical patient care in the middle of the twentieth century, as the relatively small size of the country makes it possible to obtain a bird’s-eye view of the different local developments in relation to the central forces that shaped the profession, and because the traditional geopolitical orientation of the Dutch made them particularly susceptible to international developments.^12^ Moreover, while being responsive to medical and scientific developments abroad, the Dutch neurosurgeons were not at the forefront of the international neurosurgical community. By foregrounding the work of less celebrated, and in a sense, more ordinary historical actors, I seek to divert the historical gaze away from the extraordinary contributions of world-leading neurosurgeons and sweeping universal narratives of technical progress towards the way neurosurgical patient care was negotiated at the local and national levels.^13^

## Seeking an “imperium in imperio”

In September 1936, the Dutch neurosurgeon Ferdinand Verbeek (1902-1958) wrote a letter to neurologist Bernard Brouwer (1881-1949), asking him to help facilitate a regular meeting in which the Dutch neurosurgeons could jointly discuss clinical and scientific matters under Brouwer’s intellectual guidance.^14^ In 1929, Brouwer had founded the country’s first neurosurgery ward within the neurology clinic of the academic hospital in Amsterdam, and had employed Ignaz Oljenick (1888-1981) as the first Dutch neurosurgeon. In order to prevent the outflow of patients to Brouwer’s clinic, and to uphold their own reputation, the academic hospitals in Utrecht and Groningen and the private psychiatric-neurological hospital in Wassenaar (*St.-Ursula kliniek*) had soon established their own neurosurgery wards, thereby reflecting the considerable demand for neurosurgical patient care and the fierce interhospital competition it engendered.^15^ In Utrecht, for example, the head of the psychiatric-neurological clinic, neurologist Leendert Bouman (1869-1936) had persuaded the Board of Directors of the academic hospital to recruit their own neurosurgeon, claiming that otherwise neurosurgical patients would “bypass our university.”^16^ In turn, the Board of Directors preemptively forbade the future neurosurgeon to operate in other hospitals, “in the interest of the reputation” of the academic hospital.^17^ The competition for neurosurgical patient care also unfolded within individual cities. In 1935 for instance, Verbeek had settled as neurosurgeon at the surgical clinic of the academic hospital in Groningen. Following several conflicts with his surgical colleagues, however, he resigned, and was gladly welcomed by the private Roman Catholic hospital in Groningen, where he was given an exceptional degree of clinical autonomy. Such interhospital competition even took on an international dimension through the correspondence between Dutch physicians and renowned neurosurgeons abroad. In 1931, for instance, Brouwer wrote to Cushing that there was no need “of extra men being planted in Utrecht, Groningen, etc., since Oljenick ought pretty well for the next several years at least to cover the Holland tumours,” aptly revealing the way in which Brouwer sought to give his own clinic a competitive advantage by preventing Cushing from training additional Dutch neurosurgeons.^18^

The endorsement of neurosurgery by the Dutch hospitals was generally shared by the medical community, as most neurologists and general surgeons had come to subscribe the need for a specialist exclusively dedicated to nervous system surgery.^19^ The general acceptance of neurosurgery by the medical community also found expression in the specialty’s formal recognition by the Dutch Medical Association in 1938, upon which the neurosurgeons could be formally registered by the Specialist Registration Committee (*Specialisten Registratie Commissie*).^20^ Yet even though the *raison d’être* of neurosurgery was no longer subject of significant debate, its fate as an autonomous medical specialty had all but been decided. Similar to Amsterdam, the neurosurgery wards in Utrecht and Wassenaar had been physically and institutionally embedded within psychiatric-neurological clinics that were headed by neurologists, thereby effectively subordinating the neurosurgeons to neurological leadership. During the 1930s, this state of affairs was not explicitly contested by Dutch neurosurgeons, not in the least because their neurological seniors had facilitated their appointments and had much stronger ties with the local authorities. But there was also a general sense among the neurosurgeons that they needed the clinical and scientific expertise of their neurological patrons to guide their neurosurgical practice. Indeed, in November 1936, Brouwer complied with Verbeek’s request to facilitate a platform for the Dutch neurosurgeons and hosted the first meeting of the Study Club for Neuro-Surgery in Amsterdam.^21^ At the meeting, the four neurosurgeons unanimously elected Brouwer as chairman of the Study Club and agreed to admit an equal number of neurosurgeons and neurologists as members, suggesting that they did not yet see much harm in involving neurologists in their affairs.

This changed in December 1939, when the neurosurgeon Cornelis Lenshoek (1902-1969) wrote a letter to the Board of Directors of the academic hospital in Utrecht, in which he demanded that the neurosurgery ward would be recognized as an “autonomous department.”^22^ According to Lenshoek, the need for such autonomy was evident in light of the specialty’s historical development and the organization of neurosurgery in other countries. Indeed, he argued that, “at present, independent neurosurgeons are working in almost every country,” but that “certain misunderstandings” had impeded a similar course of development in the Netherlands. In the past, Lenshoek explained, neither the neurologist nor the general surgeon had carried the full responsibility for the patient undergoing nervous system surgery: the neurologist was not accountable for the surgical intervention, whereas the surgeon could not be held responsible for the diagnostic process. To Lenshoek, then, the emergence of modern neurosurgery was based on the premise that one single person had to be responsible for the neurosurgical patient. The obvious advantages of this unilateral commitment, he argued, had been clearly demonstrated by Cushing, who had always come to his diagnosis “completely independently.” Consequently, Lenshoek argued, the neurosurgeon should endeavor to master neurological diagnostics by spending “much time and energy examining his patients before and after the operation.”

Lenshoek’s letter marked the beginning of a lingering dispute with neurologist Willem Sillevis Smitt (1894-1985), who had succeed Bouman as head of the psychiatric-neurological clinic in 1936. Upon receiving Lenshoek’s letter, the Board of Directors had turned to the Board of Curators of Utrecht University, which subsequently consulted Sillevis Smitt. The neurologist objected to Lenshoek’s aspired independence, which he claimed the neurosurgeon had already started to put into practice: “Lenshoek is a surgeon, though wants to act as being a neurologist himself. […] He keeps them [patients] to himself, even when it turns out they don’t need surgery.”^23^ According to Sillevis Smit then, all patients considered for neurosurgical interventions should first be examined by the head of the psychiatric-neurological clinic (i.e., himself). This, he argued, was primarily in the interest of education, as patients who came under Lenshoek’s solitary care were no longer available for neurological patient demonstrations, thereby fueling the curators’ concerns about the quality of the medical curriculum. On a more fundamental level, Sillevis Smit believed that “in neurosurgery, the surgical indication should be established by a neurologist,” just as the internal physician usually established the indication for the general surgeon. And while Lenshoek had compared Dutch neurosurgery to the state of neurosurgery abroad to support his claim for autonomy, Sillevis Smit drew a national comparison to argue the contrary, claiming that “at none of the universities, an *imperium in imperio* [empire within an empire] exists in the way Lenshoek desires.”

Their conflicting views aside, the two specialists were also driven by financial and reputational motives. Upon Lenshoek’s appointment, the Board of Directors had allowed him to independently treat first and second class patients, an arrangement that had been made to financially compensate the neurosurgeon for restricting his practice to the academic hospital.^24^ Third class patients, on the other hand, were to be formally admitted by the head of the clinic, who would then be able to assesses whether the patients could be used for educational purposes. By the turn of the 1940s, Lenshoek had grown increasingly displeased with this arrangement, feeling that his private income was unduly compromised by the exclusivity clause and the inability to independently admit and treat third class patients.^25^ Sillevis Smitt, in turn, ran the risk of being bypassed in the care for third class patients eligible to neurosurgery, a particularly pressing concern for the still relatively inexperienced neurologist, as senior neurologists in the area – reluctant to have their diagnosis checked by someone their junior – were already referring their first and second patients directly to Lenshoek, thereby denying the neurologist an important source of income and undermining his clinical authority.^26^

In February 1940, after hearing all parties involved, the Board of Directors drafted an “Instruction for the Neuro-Surgeon,” a two-page document outlining the rights and duties of the neurosurgeon in relation to the head of the psychiatric-neurological clinic.^27^ The Instruction simply reiterated the conditions stipulated by the Board upon Lenshoek’s appointment: the neurosurgeon was allowed to independently treat first and second class patients in the neurosurgery ward, but was strictly forbidden to practice outside of the academic hospital. Third class patients were assigned to the head of the clinic, even when they had been directly referred to the neurosurgeon by outside neurologists. The ineffectual document aptly reflected the complex dilemma the Board of Directors was facing, on the one hand trying to cater to needs of their neurosurgeon, who seemed to be increasingly aware of his strong bargaining position as one the few neurosurgeons in the country, while also maintaining their competitive edge over other hospitals and taking into account the interests of medical education. This may explain why the document was evidently based on strategic considerations rather than any specific vision on the optimal clinical relationship between neurologists and neurosurgeons.

The “Instruction for the Neuro-Surgeon” proved unworkable. Lenshoek still refused to involve Sillevis Smitt in the care of third class patients who had been directly referred to him by outside neurologists, while Sillevis Smitt continued to insist that Lenshoek should not be allowed to treat any patients in the neurosurgery ward without his involvement.^28^ Even though the Board of Directors initially stood firm, the situation changed when in May 1940, Ignaz Oljenick was forced to flee the country right before German troops invaded the Netherlands.^29^ This left the neurology clinic in Amsterdam without a neurosurgeon, a problem that Brouwer intended to solve by recruiting Lenshoek. In an attempt to keep Lenshoek for Utrecht, the Board of Directors consulted the Ministry of Education, Arts, and Sciences, which bounced the problem back to the local level by tasking the Board of Curators to mediate among Lenshoek, Sillevis Smitt, and the Board of Directors.^30^ The curators, however, sided with Sillevis Smitt, arguing that, contrary to neurology, neurosurgery was not part of the medical curriculum, and that as such, the professor of neurology should be in charge of all patients at the *academic* clinic.^31^ Indeed, the curators maintained that “it cannot be expected of future doctors that they learn the technique of nervous system surgery,” whereas “making a diagnosis and establishing an indication for surgical intervention” were essential aspects of medical education that clearly belonged to the domain of neurology. For the curators, then, neurosurgery was an exclusively surgical endeavor, fundamentally different from clinical diagnostics.^32^

Yet despite their support for Sillevis Smitt, the curators realized that Lenshoek’s imminent departure for Amsterdam would inevitably lead to the closure of the neurosurgery ward in Utrecht, as trained neurosurgeons were hard to come by. This the Board of Curators, the Board of Directors, and the Ministry all felt would seriously harm the reputation of the academic hospital and the University. Hence, curators and hospital administrators worked out an alternative proposal, in which Lenshoek would divide his time between the neurology clinic in Amsterdam and the psychiatric-neurological clinic in Utrecht.^33^ But Lenshoek had already made up his mind: he accepted Brouwer’s offer and left for Amsterdam, where in contrast to Utrecht, he was allowed to treat private patients in other hospitals.^34^ The foundation of an *imperium in imperio* would have to wait, as in Brouwer’s clinic Lenshoek would continue to work under the clinical supervision of his neurological colleague.

## Diagnosticians in Distress

Clearly, to strengthen their jurisdictional claim over the neurosurgical patient, neurosurgeons and neurologists both had to convince external stakeholders of their legitimate involvement in neurosurgical patient care. Such public efforts at persuasion typically centered around the issue of diagnostics, for which both types of specialists attempted to take problem ownership.^35^ Since the late-nineteenth century, neurologists had fashioned themselves as diagnosticians, and had developed an elaborate diagnostic method – the neurological exam – that seamlessly integrated neurophysiological insights and clinical observations to come to a scientific classification of nervous system disorders.^36^ Moreover, while the practices of neurology and psychiatry were still intertwined, particularly in the non-academic setting where “neuropsychiatrists” continued to treat both “organic” and “functional” disorders, the fields had increasingly drifted apart in the academic context.^37^ In this separation process, psychiatrists had by and large assumed problem ownership of psychotherapy, thereby withdrawing a potent therapeutic intervention from the neurologists. Even though neurologists in the Netherlands had achieved considerable institutional and academic authority, they were well aware of the precarious state of their specialty.^38^ The growing tendency of neurosurgeons to venture into the diagnostic territory, then, posed yet another serious threat to their jurisdiction.^39^ The rise of invasive diagnostic technologies for the diagnosis and localization of nervous system disorders further augmented this threat, as it gave the neurosurgeons an opportunity to draw the diagnostic process within their own jurisdictional domain, the common and effective strategy of abstraction conceptualized by Abbott.

In November 1940, Utrecht neurologist Hendrik Stenvers (1889-1973) launched an attack on his neurosurgical colleagues in the Dutch Journal of Medicine (*Nederlandsch tijdschrift voor geneeskunde*). Stenvers had been troubled by the neurosurgeons’ increasing use of ventriculography, an invasive method to diagnose and localize lesions within the skull through the injection of air into the ventricles of the brain, and seized the opportunity to persuade the medical community of the imminent dangers of leaving the neurosurgeons unchecked without firm neurological guidance.^40^ According to Stenvers, neurologists and neurosurgeons fundamentally differed in character and working method. While neurology appealed to individuals with “a more or less contemplative lifestyle, who are able to wait until synthesis arises from a multitude of facts,” neurosurgery attracted those with an “urge for action,” always willing to intervene when the opportunity presented itself.^41^ It therefore came as no surprise, the neurologist argued, that “a simple and primitive tool with great practical value” such as ventriculography, was so enthusiastically adopted by his neurosurgical colleagues.^42^ For Stenvers, this uncritical use of ventriculography was detrimental to the future progress of neurology, as it replaced the scientifically-grounded neurological exam with “a purely mechanical tool that requires little or no cognitive effort and lacks any insight into the workings of the nervous system.”^43^ “The more ventriculographies, the less neurology,” Stenvers summarized his view, and concluded by praising “the fortunate situation that […] neurosurgery is practiced in very intensive and meticulous collaboration between neurologist and neurosurgeon.”^44^

Stenvers’s article instigated a discussion with the neurosurgeons De Vet and Verbeek, who claimed that ventriculography was an invaluable diagnostic tool that should be called upon very often, precisely because “the neurological examination does not provide the neurosurgeon with the exact information necessary or desirable for his operation.”^45^ “Which neurosurgeon […] would want to work in such diagnostic mist?” Verbeek bluntly remarked, thereby framing the neurosurgeons’ resort to ventriculography as a necessary consequence of the neurologists’ diagnostic shortcomings.^46^ In addition, Verbeek sought to disqualify his neurological colleagues by ridiculing their contemplative attitude, accusing Stenvers of being a sentimentalist, who could not accept that “the romantic ways of neurology” had to make way for nothing but “air.”^47^ Neurosurgeons, he continued, were indeed more pragmatic, but not because they desperately longed for action but because they prioritized “the cure or even the *relief* of *suffering* of every patient” over “the progress of neuro-physiology.”^48^ Indeed, by accusing neurologists of single-mindedly pursuing scientific progress at the expense of their patients, Verbeek effectively reframed the neurosurgeons’ pragmatism and therapeutic readiness as altruistic identity traits in order to claim moral superiority over his neurological colleagues.^49^ And as for the “intensive and meticulous” collaboration between neurosurgeons and neurologists that Stenvers propagated, Verbeek left no doubt about his views on the matter, arguing that in the past, surgeons had always “stood by their contemplative neurological colleagues and had catered their every need,” which had resulted in nothing but “troublesome mortality and much professional resentment.”^50^

In January 1942, the neurologists launched a second campaign to defend their diagnostic jurisdiction, this time in the form of a public lecture on neurological diagnostics by Brouwer, who had been appointed President (*Rector Magnificus*) of the University of Amsterdam around the start of the German occupation.^51^ In addressing the academic community and local authorities of Amsterdam, Brouwer struck a more conciliatory tone than Stenvers had done in the *Dutch Journal of Medicine*, generously recognizing the imperfections of the neurological exam and acknowledging the neurosurgeons’ desire to refine the diagnostic process.^52^ Yet despite being a “powerful diagnostic tool,” Brouwer argued that the uncritical use of ventriculography caused physicians to lose sight of the human aspect of the diagnostic process, thereby leading them to wrong conclusions about the patient’s illness.^53^ The neurological exam, by contrast, addressed these human aspects, as neurologists were skilled in translating patients’ subjective experiences into an objective understanding of disease. Moreover, since ventriculography was occasionally accompanied by serious complications, Brouwer maintained that its use should be restricted to the rare cases in which the neurological exam remained inconclusive. To support this claim, Brouwer had reviewed all his brain tumor cases of the previous year. In the majority of cases, he explained, the right diagnosis and localization had been made based on the neurological exam alone, and of the patients who had been exposed to ventriculography, the procedure had only provided important new diagnostic insights in about one third of the cases. “The numbers speak,” Brouwer concluded, numbers that allegedly demonstrated the undisputed value of the neurological exam as the principal method of diagnosing and localizing nervous system disorders.^54^

**Figure 1:**
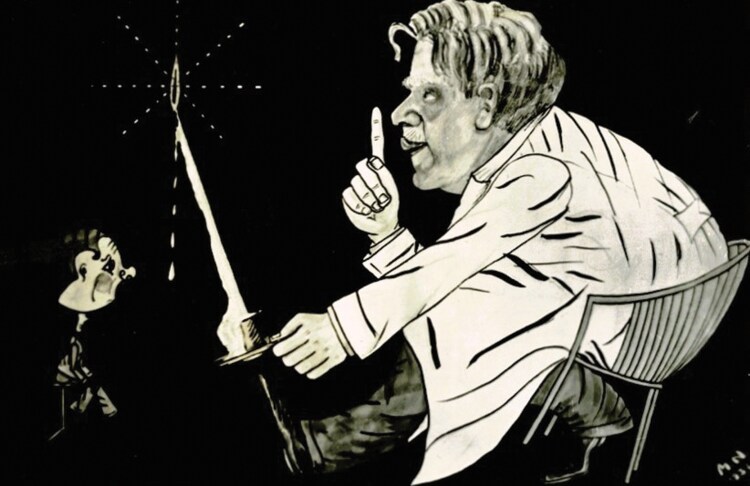
Caricature of neurologist Bernard Brouwer silencing his students as President of the University of Amsterdam. Archives NVvN.

The following day, Brouwer’s lecture was featured in the *National Daily* (*Het Nationale Dagblad)*, a newspaper distributed by the Dutch National Socialist Movement.^55^ The reporter singled out the “eminent significance” of the interaction between doctor and patient in the diagnostic process as its most important message, thereby propagating Brouwer’s depiction of the neurologist as a guardian of humanity in the face of growing mechanistic threats. Indeed, Brouwer’s role as President of the University of Amsterdam imbued him with substantial public and institutional authority, which certainly helped him to promote the neurologists’ cause. But his prominent wartime position also forced Brouwer to cooperate with the Nazi regime, which had prompted several students to accuse him of treason.^56^ In September 1942, Brouwer publicly responded to these accusations, stating that he preferred students who “stayed silent” over those who voiced their disapproval, a demeaning remark that subsequently formed the content of a cartoon, in which an inflated representation of Brouwer was depicted as silencing an evidently marginalized student from his privileged position in a comfortable chair [see Figure 1].^57^ Nevertheless, Brouwer felt that it was his duty preserve the university “for better or for worse,” and expressed his regret that his students would not see “that it is the captain’s job to sail in the storm” and did not appreciate “the tactical maneuvering of their Rector.”^58^ These comments aptly exposed Brouwer for the seasoned strategist he was, a quality that was not restricted to university politics but also came to expression in his role as chairman of the Study Club for Neuro-Surgery, where he sought to tame the gathering storm between neurologists and neurosurgeons.

## “Neurological and Neuro-surgical talents and temperaments”

In November 1946, at the occasion of the tenth anniversary of the Study Club for Neuro-Surgery, Verbeek reflected on the many discussions between neurologists and neurosurgeons over the preceding decade. Although these discussions had always been carried out “in a courteous manner,” the “Feu Sacrée [*sic*]” had certainly been present, since “in these discussions our Neurological and Neuro-surgical talents and temperaments become most apparent.”^59^ From the outset, the meetings of the Study Club centered around the exchange of medical and scientific insights amongst the “official” Dutch neurosurgeons and a select group of elite neurologists. Such an “exclusionist ethos” allowed the neurosurgeons with extensive neurosurgical training to set themselves apart from general surgeons who were still practicing nervous system surgery.^60^ In addition, restricting neurological membership enabled the neurological members to frame the diagnosis of patients eligible to neurosurgery as a highly demanding and exclusive branch of neurology, providing them with a competitive advantage over their less prominent colleagues. Such restriction efforts seemed particularly urgent, since the neurosurgical and neurological members of the Study Club both feared that the establishment of more neurosurgery wards in the country would oversaturate the market for neurosurgical patient care.^61^

**Figure 2:**
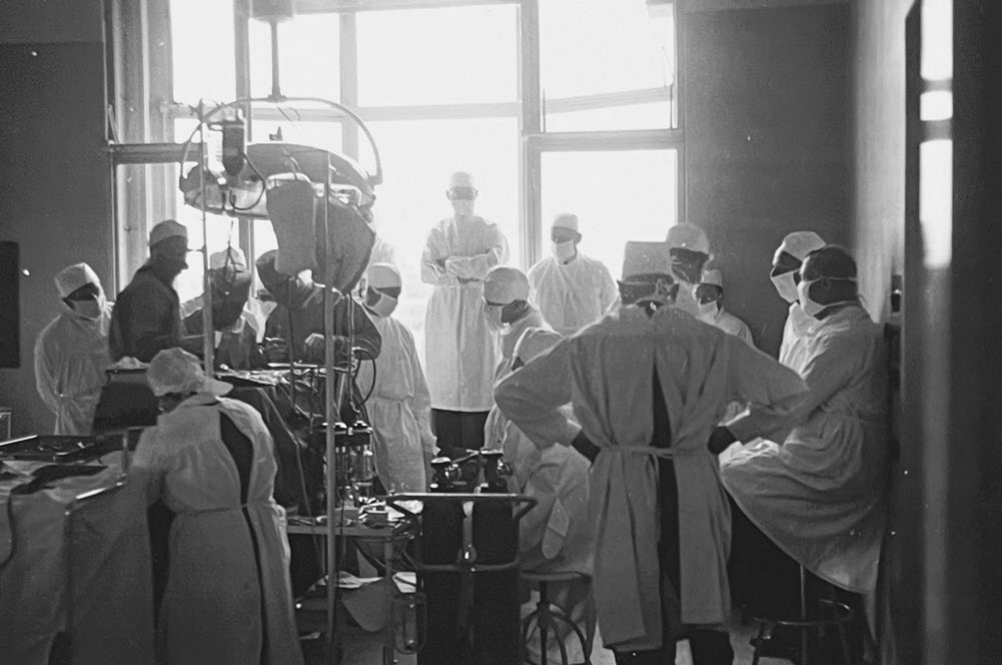
Neurosurgical demonstration at a meeting of the Study Club for Neuro-Surgery, ca. 1950. Archives NVvN.

While the meetings of the Study Club initially consisted of two-hour presentation-discussion sessions hosted at one of the four neurosurgery wards in the country, the introduction of surgical demonstrations in 1940 turned the meetings into full day events. In the morning, the host neurosurgeon performed one or two operations while the other members of the Study Club crammed together in the operating room to watch the performance [Figure 2]. These demonstrations allowed the neurosurgeons to exchange tacit knowledge – such as particular ways of moving and handling surgical instruments – and to act out their therapeutic pragmatism by flaunting their steady hands and quick reflexes for an audience of neurologists outside of their natural habitat.^62^ The surgical demonstrations were followed by presentations in the afternoon, in which neurosurgeons usually elaborated on the technical details of the procedure and neurologists reflected on the diagnostic process. During these presentations, both types of specialists employed interactive and visual strategies, such as live patient demonstrations and the display of photographs, radiograms, and pathological specimens, to engage the audience, support their claims, and demonstrate their professional expertise.^63^ Under Brouwer’s firm leadership, the subsequent discussions never digressed into serious contestation, even though they did occasionally become quite spirited when touching upon contested jurisdictional domains, such as the question whether or not to operate certain patients based on specific diagnostic findings. Such discussions created considerable tensions, which were reinforced by the various unspoken conflicts between individual neurological and neurosurgical members outside of the Study Club. To this end, the dinner parties that were organized by one of the hosting members at the end of the day – where “personal squabbles were smoothed out in a pleasant way” through the exchange of “not so chaste jokes and stories over drinks and at the dinner table,” which had allegedly earned some members “legendary fame” – served to diffuse underlying tensions through humor and shared masculinity.^64^

From the early 1940s onwards, however, the accumulation of professional issues increasingly put strain on the friendly relations among members of the Study Club. Such issues mainly involved the formulation of neurosurgical training requirements, a topic that had first surfaced in the late 1930s, when the Specialist Registration Committee had asked Brouwer whether the primary focus of the neurosurgeon’s training should be on neurology or surgery.^65^ Particularly, the question if and to what extent future neurosurgeons should receive training in neurology was subject of serious debate. Neurologists such as Brouwer argued that neurology should not be an obligatory part of neurosurgical training, since a better grasp of neurology would encourage neurosurgeons to move further into diagnostic territory.^66^ Precisely for this reason, neurosurgeons pleaded for the opposite and demanded more neurological training to enhance their diagnostic expertise and expand their clinical autonomy. Nevertheless, neurologists and neurosurgeons both contended that the neurosurgeon should primarily be a surgeon rather than a neurologist. This became readily apparent when in 1942, Utrecht neurosurgeon Henk Verbiest (1909-1997) was considered for membership of the Study Club. Verbiest had been fully trained as a neurologist prior to commencing his neurosurgical training abroad, and this raised the fundamental question whether someone with a neurological background and predisposition could become a proper neurosurgeon.^67^ Brouwer, for instance, argued that “it is better when special surgeons do this work in intimate collaboration with neurologists” rather than neurologists taking up neurosurgery themselves.^68^ And Verbeek scathingly remarked that “these specialists can be of no more value to medicine than unviable double monsters [conjoined twins], too little of twins and too deformed to be one.”^69^ Unsurprisingly then, Verbiest’s membership was initially rejected, much to the dismay of Sillevis Smitt, who had hired Verbiest on Lenshoek’s former position at the academic hospital in Utrecht and wanted his new neurosurgeon to be recognized by the Study Club in order to revive his clinic’s reputation.^70^ After two years, however, the members of the Study Club could no longer justifiably deny Verbiest membership, since despite his neurological background, he eventually fulfilled all the neurosurgical training requirements and achieved good surgical results, forcing the members of the Study Club to admit that there were indeed neurologists who could subsequently become neurosurgeons.

**Figure 3:**
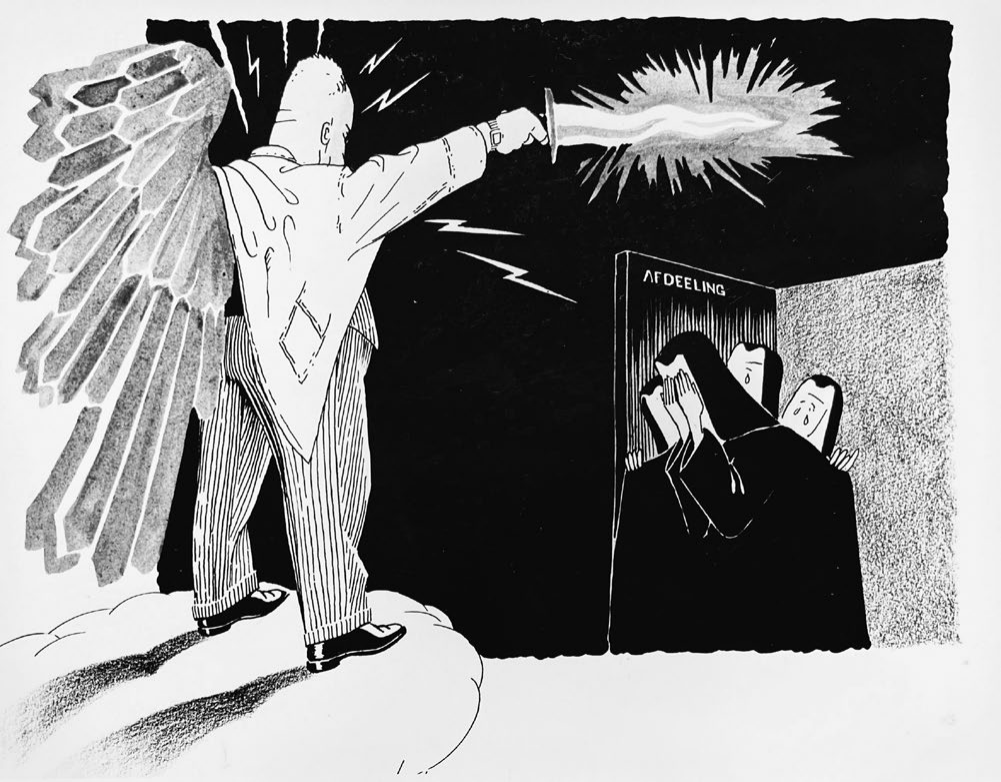
Neurologist Eduard Hoelen depicted as divine ruler of the St.-Ursula Clinic in Wassenaar. Piet van der Maaden, 1943, Archives NVvN.

In dealing with the various professional issues that surfaced during the 1940s, Brouwer always remained firmly in charge. As chairman of the Study Club, nearly all external communications went through him, and in case of disagreement among members, the neurologist usually had the final say.^71^ Indeed, while reflecting on Brouwer’s leadership style, De Vet later recounted, “There was old-fashioned order and discipline. […] discussions took place in a strict hierarchical order, where the right to speak was exclusively granted by the chairman,” and “when one of the members missed a winter meeting because he had had the courage of going on a ski trip, he was unmistakably informed that this should not happen a second time.”^72^ Brouwer ran a similar authoritarian regime in his clinic in Amsterdam where he firmly pulled the strings, and according to Verbiest, once remarked that the neurosurgeon’s “hands” were but an extension of the neurologist’s “brains.”^73^ At the other Dutch psychiatric-neurological clinics that hosted a neurosurgery ward, neurologists had similarly managed to consolidate their leadership, thereby securing their involvement in neurosurgical patient care. This absolute rulership of the neurologists was aptly depicted by the Dutch illustrator Piet van der Maaden (1919-2007), who was in hiding at the St.-Ursula Clinic in Wassenaar during WWII, and in the numerous cartoons he made about the daily life at the psychiatric-neurological hospital frequently portrayed neurologist Eduard Hoelen (1896-1962) as the divine ruler of the clinic [Figure 3].^74^

**Figure 4:**
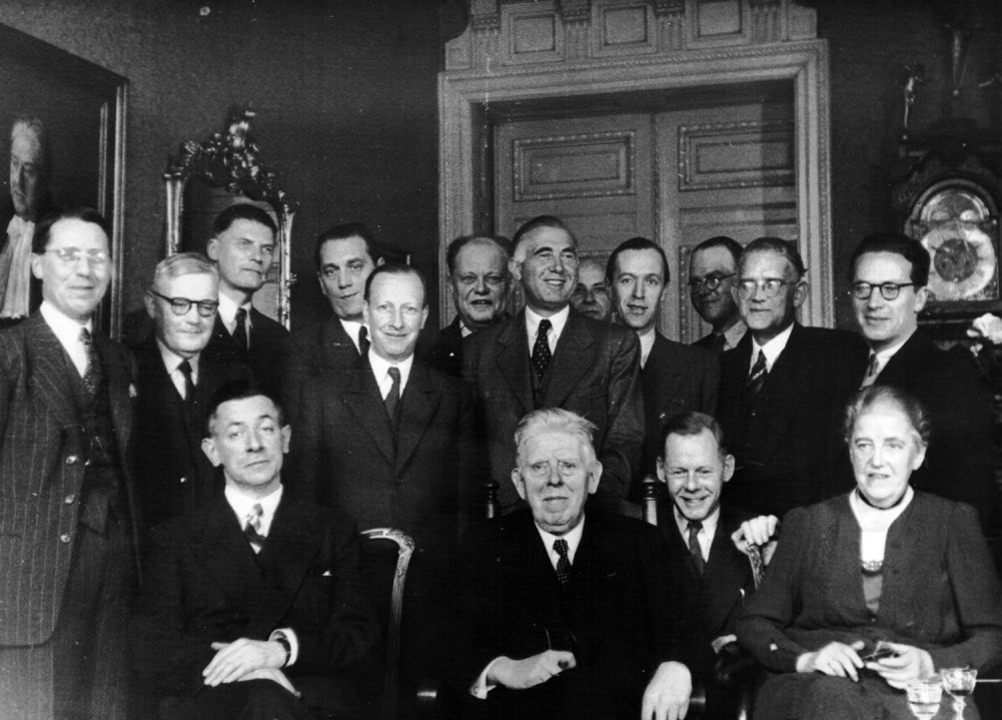
Group photo taken at the tenth anniversary of the Study Club for Neuro-Surgery, 9 November 1946. Standing (L-R): Joseph Prick, Hendrik Stenvers, Willem Sillevis Smitt, Henk Verbiest, Bernard Ziedses des Plantes, Eduard Hoelen, Arnold De Vet, Melle Weersma, Paul Hoeberechts, Shell de Grood, Arie Biemond, Paul Hanraets. Sitting: Ferdinand Verbeek, Bernard Brouwer, Willem Noordenbos Jr., Hélène Marie Brouwer-Frommann. Archives NVvN.

But the tables were turning. Following the Dutch liberation, Brouwer was publicly accused of collaborating with the Nazi regime during his time as President of the University of Amsterdam and was subsequently discharged as head of the neurology clinic in April 1946.^75^ Even though Brouwer did not become a *persona non grata* – the medical and academic communities were generally understanding of his difficult position during the war and he was soon offered a new position as Director of the Central Institute for Brain Research (*Centraal instituut voor hersenonderzoek*) – his public loss of face and waning institutional power destabilized the Dutch neurologists’ authority, both within and outside of the Study Club. And while Brouwer stayed on as chairman of the Study Club, his control over the Dutch neurosurgeons began to weaken. Thus in November 1946, at his speech at the tenth anniversary of the Study Club, Verbeek still praised Brouwer as “the great promoter and driving force in the neurosurgical field” and acknowledged the friendly relations among members of the Study Club – optimistic remarks that seemed to be supported by the cheerful group photo that was taken at the occasion [Figure 4]. Yet Verbeek ended his speech on an unprecedented critical note, stating that “there is still stark opposition regarding many important neurosurgical issues,” thereby preluding the reinvigorated quest for independence to which the neurosurgeons were about to embark.^76^

## Throwing Off the Shackles

**Figure 5:**
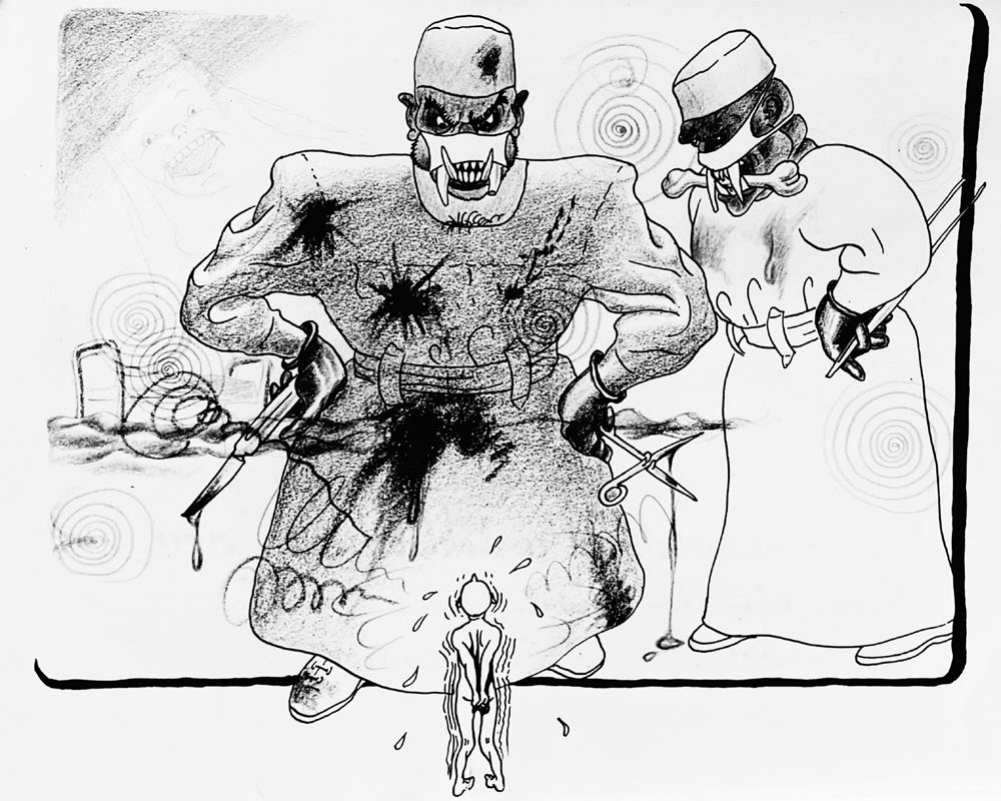
Neurosurgeons depicted as terrifying monsters. Piet van der Maaden, 1943, Archives NVvN.

The neurosurgeons’ final tour the force was set in motion by the specialty’s imminent academic recognition. During the 1940s, the demand for neurosurgical patient care had further increased, prompted by the rapid emergence of novel therapeutic procedures, most notably the rise of herniated spinal disc surgery and the increased accessibility to healthcare.^77^ According to Verbeek, the “satisfactory results of the first pioneering years” had also contributed to the “widespread confidence in the art of neurosurgery,” particularly among patients, who he argued increasingly insisted on neurosurgical inventions.^78^ Another cartoon by Piet van der Maaden, however, in which the artist-in-hiding depicted the neurosurgeons at the St.-Ursula Clinic as savage bloodthirsty monsters towering over a terrified patient, revealed that there was a different side to the story as well [Figure 5].^79^ Indeed, many neurosurgical interventions were still associated with substantial risks and complications, making at least some patients feel extremely vulnerable and fearful when facing a neurosurgeon. Even so, the rapid expansion of neurosurgery did accelerate the competition for neurosurgical patient care among hospitals and universities throughout the country, and under influence of these competitive forces, the first opportunity for neurosurgery’s academic recognition eventually arose in Groningen.

The academic hospital in Groningen had been left without a neurosurgeon since Verbeek had resigned from his position at the surgical clinic in 1936. Although the neuropsychiatrist Willem van der Scheer (1882-1957) had subsequently sought to establish a new neurosurgery ward within the psychiatric-neurological clinic, the war had temporarily put the plans on hold. This situation, the Board of Curators felt, “could not be sustained,” as patients eligible to neurosurgery had to be referred to other clinics, thereby harming the reputation of the academic hospital.^80^ Thus following the liberation, the curators, in consultation with Van der Scheer, had appealed to the Ministry of Education to ask permission for the foundation of a neurosurgery ward within the psychiatric-neurological clinic. In addition, the curators requested financial support for the training and subsequent appointment of a neurosurgeon at the rank of *lector*, a prominent academic position that would bolster the prestige of the new neurosurgery ward.^81^ The Ministry tentatively approved the requests, and financed the final training stage of aspiring neurosurgeon Arie van der Zwan (1907-1999) in England, indicating that, at least initially, the government had no serious doubts about neurosurgery’s academic legitimacy.^82^

When Van der Zwan returned from his neurosurgical training in England and applied to be registered by the Specialist Registration Committee, the news about his imminent appointment as lector in Groningen sent a shockwave through the Study Club. The neurosurgeons were the first to protest. Verbeek argued that Van der Zwan’s training had been inadequate, claiming that the two years Van der Zwan had spent at the neurosurgical ward at the Roman Catholic hospital could not count as neurosurgical training, “since, against my advice, Dr. van der Zwan never took notes of my patients, neither before nor after the operative procedure.”^83^ And De Vet maintained that “this [neurosurgical] lectorate can hardly be assigned to someone who has never independently practiced neuro-surgery.”^84^ For once, the neurologists agreed with their neurosurgical colleagues, albeit for different reasons. Whereas the neurosurgeons merely condemned the appointment of an inexperienced junior at a prestigious academic position, the neurologists disapproved of the academic independence of neurosurgery in general, although they were smart enough to keep this to themselves. Thus, despite their divergent motives, the members of the Study Club agreed to advise the Specialist Registration Committee to reject Van der Zwan’s application until he had fulfilled a year of additional training.

Van der Zwan’s application was indeed rejected, and upon receiving the news, the Board of Curators of the University of Groningen asked the Ministry to suspend his appointment. In the meantime, the Curators of Utrecht University also requested the Ministry to promote their neurosurgeon to the rank of lector, as they feared that the academic hospital in Groningen would try to recruit Verbiest by offering him Van der Zwan’s lectorate.^85^ In consultation with Sillevis Smitt, however, the Medical Faculty in Utrecht had urged the Board of Curators that “it is not the intention to establish a lectorate in neuro-surgery,” but to merely to grant Verbiest the personal title of lector, thereby perpetuating his subservience to the psychiatric-neurological clinic.^86^ But the whole course of events had raised concerns with the Ministry. While the Minister saw no harm in promoting Verbiest to lector on a personal title in Utrecht, Van der Zwan’s appointment in Groningen was more problematic, not only due to the objections raised by the members of the Study Club, but also because the Ministry had started to question the desirability of burdening the medical faculties with the manifestation of yet another specialty.^87^ All in all, the Dutch neurosurgeons had much to criticize about the way their neurological colleagues had handled their pending academic recognition. In Groningen, Van der Scheer’s nomination of an immature candidate had seemingly ruined their chances for academic independence, while in Utrecht, Sillevis Smitt had obstructed the foundation of an independent academic position by promoting Verbiest to lector on a personal title. Finally then, the neurosurgeons had reached a breaking point and decided to take matters into their own hands.

Thus in October 1948, the Dutch neurosurgeons jointly wrote a letter to the neurological members of the Study Club in which they demanded the “complete and exclusive leadership” of the neurosurgery wards in the country, thereby drawing on familiar rhetorical strategies, such as the glorification of Cushing, denouncing the past division of labor between neurologists and surgeons, and contrasting the organization of neurosurgery in the Netherlands to the state of the specialty abroad.^88^ The letter infuriated some of the neurological members of the Study Club, including Joseph Prick (1909-1978), who claimed that granting the neurosurgeons clinical autonomy would cause them to move further into diagnostic territory, thereby putting “every patient with headache or back pain at risk of being exposed to ventriculography and myelography [respectively].”^89^ The neurologist Arie Biemond (1902-1973), Brouwer’s successor as head of the neurology clinic in Amsterdam, on the other hand, admitted that resisting the neurosurgeons’ “remarkable urge for living space” would be “a counterproductive strategy.”^90^ Hence, he proposed to alleviate the “disputed hegemony” by allowing the neurosurgeons to independently treat some patients within the (psychiatric-)neurological clinics.^91^ Even though this arrangement would still keep the neurosurgeons confined to the neurological clinic, they reluctantly consented, after Brouwer, who also acknowledged the need for compromise to avert an imminent break with his neurosurgical colleagues, expressed himself in favor of the proposal.^92^

But despite this provisional compromise in the Study Club, the neurosurgeons continued their quest for independence in the public realm. At the close of 1948, the Ministry finally decided to proceed with Van der Zwan’s appointment as lector in Groningen, even though the Minister stressed that neurosurgery should not consequently become part of the medical core curriculum, and as such, did not need to be fully represented at all of the Dutch universities.^93^ Clearly, the government support for neurosurgery was approaching its limits, indicating that there was still a significant amount of lobbying to be done for the Dutch neurosurgeons. In 1949 then, Van der Zwan and Verbiest both delivered public lectures upon accepting their lectorates, in which they each adopted their own strategy to promote the clinical and institutional independence of neurosurgery. Van der Zwan used a straightforward approach, making “Neuro-surgery as Independent Specialty” the title of his lecture, listing all the countries where neurosurgery had obtained an autonomous status, and going to great lengths to show how Cushing’s legacy had made the independence of neurosurgery inevitable.^94^ Verbiest’s adopted a different tactic, strongly positioning himself as a thoughtful, self-critical and cultivated man of science, and highlighting the many ways in which neurosurgery could contribute to neuroanatomical and neurophysiological knowledge.^95^ In doing so, Verbiest mobilized his dual identity as neurologist to propagate a “new” physiological type of neurosurgery – “a surgery of neuroregulation” – that replaced the crude macroscopic brain resections of the past for highly precise, scientifically-grounded interventions aimed at restoring the patient’s functional balance.^96^ Indeed, by propagating a functional approach to neurosurgery, Verbiest was able to realign his specialty to the holistic tendencies that permeated biomedicine at the time, thereby boosting his specialty’s scientific legitimacy and countering the pervasive image of the neurosurgeon as unthoughtful man of action.^97^

Following the lectures of Van der Zwan and Verbiest, the neurologist Prick embarked on a public countercampaign. In an article published in the Roman Catholic Medical Journal (*R.K. Artsenblad*), Prick claimed that “even though in America and some Europeans countries, neurosurgery was born from general surgery – in our country her obstetrician was a neuropsychiatrist and she remains a legitimate child of neurology.”^98^ The Dutch neurologists, he argued, had primarily been responsible for the development of neurosurgery through their scientific and diagnostic contributions, and should sustain their control over the field to prevent neurosurgery from digressing into “a purely technical endeavor, with all the resulting deficiencies.”^99^ But despite Prick’s rebuttal, the last neurological defenses were torn down when, in November 1949, Benard Brouwer, the neurologist who had been instrumental in keeping the neurological and neurosurgical members of the Study Club together, suddenly passed away. In his eulogy delivered at the next meeting of the Study Club, Lenshoek evoked a particular interpretation of Brouwer’s legacy to further the neurosurgeons’ cause, claiming the posthumous blessing of Brouwer by arguing that the neurologist had “strongly stimulated our [the neurosurgeons’] quest for independence,” and that the Dutch neurosurgeons would “continue to honor him by pursuing this quest ever more vigorously.”^100^

In the period that followed, the neurological and neurosurgical members of the Study Club increasingly grew apart as jurisdictional conflicts continued to accumulate. In dealing with these conflicts, the Dutch neurosurgeons more often convened in private, no longer entrusting their neurological colleagues with their professional affairs. In a desperate attempt to avoid the inevitable, the neurologists invited the neurosurgeons to form a subdivision of the Netherlands Society for Psychiatry and Neurology. Unsurprisingly, the neurosurgeons declined the request, even though they realized that they needed the formal backing of a professional society to strengthen their bargaining position. Indeed, without the support of such society, they were easily dismissed by the authorities. In 1952, in the wake of a dispute between neurologist Bernard Ziedses des Plantes (1902-1993) and neurosurgeon Melle Weersma (1902-1976) in Rotterdam, the Dutch neurosurgeons jointly wrote to the Director of the Municipal Hospitals in Rotterdam to demand more clinical and institutional autonomy for their neurosurgical colleague.^101^ They were, however, dismissed by the hospital director, who sharply responded that the municipal council of Rotterdam did not “conceive its relationship with the Dutch neurosurgeons in such a way that demands can be made,” and that the neurosurgeons should cultivate some more “souplesse d’esprit [flexibility of mind],” as there were many other emerging specialties that were placing demands on the municipal hospitals.^102^ But for the Dutch neurosurgeons there was no more “souplesse d’esprit,” and in November 1952, they informed the neurological members of the Study Club about the foundation of the Netherlands Society of Neurosurgeons (*Nederlandse Vereniging van Neurochirurgen)*, a professional society exclusive to neurosurgeons which would henceforward allow them to represent their own professional interests.^103^

## Conclusion

In October 1956, upon his inauguration as professor in neurosurgery at the University of Groningen, Cornelis Lenshoek playfully recounted the Dutch neurosurgeons’ struggle for independence.^104^ Cleverly playing on the parent-child metaphor that had been evoked by the Dutch neurologists in the past, he remarked,

Surgery and neurology have been responsible for the birth of the young specialty. As parents, they can only rejoice at the enormous growth and drive of their young sprout. That this sprout has sometimes rebelled against his parents, of whom one has neglected and the other has patronized him, is a normal phenomenon. Now the lad has grown up, however, he will strive for harmony with the elders and will not only appreciate their interest, but will also urgently need it.^105^

Indeed, Lenshoek had lived through neurosurgery’s entire coming of age journey, a process that was characterized by a hegemonic relationship between neurologists and neurosurgeons.

In the Netherlands, neurologists had been primarily responsible for the foundation of the neurosurgery wards within the neurological and psychiatric-neurological clinics, which enabled them to clinically and institutionally subordinate neurosurgery to neurology, and to exert an extraordinarily high degree of control over their neurosurgical colleagues. While the Dutch neurosurgeons increasingly strived for more clinical and institutional independence, the neurologists generally sought to impede this process in order to maintain their diagnostic authority and their field’s therapeutic potency. In line with Abbott’s conceptual framework, neurologists and neurosurgeons not only contested one another at the local workplace and at the meetings of the Study Club for Neurosurgery, but also sought to strengthen their jurisdictional claim over neurosurgical patient care in front of the outside world, thereby defining the diagnosis of nervous system disorders as a problem within their own domain (i.e., abstraction). This multilayered demarcation battle was shaped by pervasive international scientific and political developments, intense national competition among hospitals and universities, and divergent views and interests of the many different external stakeholders involved. In this process, the clear distinctions among local, national, and international forces and actors increasingly dissolved to give rise to the particular organization of Dutch neurosurgery in the middle of the twentieth century. Even though the neurosurgeons ultimately broke with their neurological patrons, the two types of specialists would continue to negotiate neurosurgical patient care, but increasingly as equal partners.

## Footnotes

1 Arnold de Vet to Ferdinand Verbeek, 29 November 1950, Box 1, Notulen 1936-1949/Correspondentie 1936-1990 Studieclub voor Neuro-chirurgie, Archives of the Netherlands Society of Neurosurgeons (*Nederlandse Vereniging van Neurochirurgen*; *NVvN*), Trefpunt Medische Geschiedenis Nederland, Urk, the Netherlands (hereafter cited as Archives NVvN), double underlined in the original.

2 In his classic comparative study of medical specialization, medical historian George Weisz has described how specialization increasingly became a matter of national regulation during the first half of the twentieth century. See George Weisz, *Divide and Conquer: A Comparative History of Medical Specialization* (Oxford: Oxford University Press, 2006), 85–187.

3 For the concept of boundary work, which was developed by sociologist Thomas Gieryn to analyze the shifting cultural boundaries between science and non-science, see Thomas F. Gieryn, *Cultural Boundaries of Science: Credibility on the Line* (Chicago: University of Chicago Press, 1999). The concept was subsequently adopted by other scholars, particularly in the field of Science and Technology Studies, who used it in a more encompassing way to analyze the battles for authority amongst groups and actors within the scientific community. See for instance, Sheila Jasanoff, *Science at the Bar: Law, Science, and Technology in America* (Cambridge, MA: Harvard University Press, 1995). In line with these broader applications, I use the term “boundary work” to indicate the various efforts by Dutch neurologists and neurosurgeons to defend and expand their bordering jurisdictional territory, often by rallying outside stakeholders.

4 Delia Gavrus, “Men of Dreams and Men of Action: Neurologists, Neurosurgeons, and the Performance of Professional Identity, 1920-1950,” *Bulletin for the History of Medicine* 85 (2011): 57–92.

5 Delia Gavrus, “Skill, Judgement and Conduct for the First Generation of Neurosurgeons, 1900–1930,” *Medical History* 59 (2015): 361–378.

6 On the history of medical specialization in the Netherlands, see Alice Juch, *De medische specialisten in de Nederlandse gezondheidszorg: hun manifestatie en consolidatie, 1890–1941* (PhD diss., Erasmus University Rotterdam, 1997).

7 For the contested emergence of neurosurgery as a medical specialty in the Netherlands during the late nineteenth and early twentieth century, see Bart Lutters, “Division of Labor of a New Discipline? The Troubled Emergence of Neurosurgery in the Netherlands, 1890-1929,” *European Journal of the History of Medicine and Health* 80 (2023): 119–151.

8 Epitomizing the contributions of renowned predecessors had long been a common strategy for physicians to promote their contemporary interests. For the recurrent “reinvention” of Hippocrates from the Renaissance through the twentieth century, see David Cantor, ed., *Reinventing Hippocrates* (London: Routledge, 2001).

9 Beyond scientific and political developments, the emergence of international specialist communities and funding networks importantly shaped local and national specialist identities by cultivating “shared tastes, class assumptions, and values for scientists and doctors beyond borders.” See Stephen T. Casper, “The Currency of Consciousness: Neurology, Specialization, and the Global Practices of Medicine,” *Canadian Bulletin of Medical History* 33 (2016): 321–363, at 324–325.

10 Andrew Abbott, *The System of Professions: An Essay on the Division of Expert Labor* (Chicago: University of Chicago Press, 1988). Abbott posited his theory in the 1980s, the heyday of the sociology of professions, with other leading contributors such as Eliot Freidson and Magali Larson. Abbot’s conceptualizations have been particularly well received by medical historians. For an account of the way in which medical historians have related to the idea of profession, see John C. Burnham, *How the Idea of Profession Changed the Writing of Medical History* (London: Wellcome Institute for the History of Medicine, 1998).

11 Abbott, *System of Professions*, 20.

12 For an analysis of the geopolitical culture in the Netherlands from the sixteenth century onwards, see Friso Wielenga, *A History of the Netherlands: From the Sixteenth Century to the Present Day* (London: Bloomsbury Publishing, 2015). Before World War I, Dutch physicians were particularly susceptible to medical developments in the German-speaking countries, France, and the United Kingdom. During the interwar period, the United States increasingly became the primary “reference culture” for modern science and medicine. For the concept of reference cultures, see Joris van Eijnatten, “Beyond Diversity: The Steady State of Reference Cultures,” *International Journal for History, Culture and Modernity* 3 (2015): 1–8.

13 In a recent volume, Delia Gavrus and Stephen Casper have addressed the need to shift focus to the more unconventional stories in the history of the mind and brain sciences, for instance, by foregrounding the contributions of less renowned historical actors, in order to destabilize the grand narratives of technical progress. See Delia Gavrus and Stephen T. Casper, “Introduction: Technique, Technology, and Therapy in the Brain and Mind Science,” in *The History of the Brain and Mind Sciences: Technique, Technology, Therapy*, ed. Delia Gavrus and Stephen T. Casper (Rochester, NY: University of Rochester Press, 2017), 1–24. Gavrus has made a similar call for the history of neurosurgery, stressing the importance of “focusing not just on elite figures, but investigating the kinds of procedures on the brain and skull performed by general and provincial surgeons who may not have been prominent on the national scene” to allow “historians to shed light on the diversity of surgical commitments present at this time and to uncover a pervasive ethos that privileged surgical intervention.” See Delia Gavrus, “Opening the Skull: Neurosurgery as a Case Study of Surgical Specialisation,” in *The Palgrave Handbook of the History of Surgery*, ed. Thomas Schlich (London: Palgrave Macmillan, 2019), 435–455, 439. For the two standard works on the history of neurosurgery, see Arthur Earl Walker, ed., *A History of Neurological* Surgery (London: Williams and Wilkins Co., 1951); Samuel H. Greenblatt, T. Dagi, and Mel H. Epstein, ed., *A History of Neurosurgery in Its Scientific and Professional Contexts* (Illinois: American Academy of Neurological Surgeons, 1997).

14 Ferdinand Verbeek to Bernard Brouwer, 18 September 1936, Box 1, Notulen 1936-1949/Correspondentie 1936-1990 Studieclub voor Neuro-chirurgie, Archives NVvN.

15 Medical historian Guenther Risse has described the phenomenon of interhospital competition in the American context. See Guenther B. Risse, *Mending Bodies, Saving Souls: A History of Hospitals* (Oxford: Oxford University Press, 1999), 463–512.

16 Leendert Bouman to the Board of Curators of the Utrecht University, 28 January 1931, Catalogue Reference 59, Inventory 1283, College van Curatoren van de Rijksuniversiteit Utrecht, Utrecht Archives, Utrecht, the Netherlands (hereafter cited as College van Curatoren, Utrecht Archives).

17 Minutes of meeting Board of Directors of the Municipal and Academic Hospital in Utrecht and Head of the Department of Higher Education of the Ministry of Education, Arts and Sciences, 19 June 1940, College van Curatoren, Utrecht Archives.

18 Rob J.M. Groen, Peter J. Koehler, and Alfred Kloet, “The Role of Harvey Cushing and Walter Dandy in the Evolution of Modern Neurosurgery in the Netherlands, Illustrated by Their Correspondence,” *Journal of Neurosurgery* 118 (2013): 542.

19 A notable exception was the surgeon Pieter Michaël (1892-1985), head of the surgical clinic of the academic hospital in Groningen, who believed that neurosurgery should remain part of general surgery. See Marius Knape, *Prof. dr. P.R. Michaël: levensbeschrijving van een chirurg* (Den Haag: Stichting Prof. Michaël-van Vloten Fonds, 1988), 43–46.

20 Minutes of board meeting of the Dutch Medical Association (*Nederlandse Maatschappij ter Bevordering der Geneeskunst*), 29 May 1938, Catalogue Reference 2.19.053.01, Inventory 29, Inventaris van het archief van de Nederlandse Maatschappij tot Bevordering der Geneeskunst, National Archives of the Netherlands, The Hague, The Netherlands. For a historical overview of the Specialist Registration Committee, see L.P.H.J. Vink, *Het ontstaan en de geschiedenis van de specialisten registratie commissie* (Utrecht: Libertas Drukwerkservice, 1986).

21 Minutes of the Study Club for Neuro-Surgery, 7 November 1936, Box 1, Notulen 1936-1949/Correspondentie 1936-1990 Studieclub voor Neuro-chirurgie, Archives NVvN.

22 Cornelis Lenshoek to the Board of Directors of the Municipal and Academic Hospital in Utrecht, 18 December 1939, College van Curatoren, Utrecht Archives.

23 Minutes of meeting Willem Sillevis Smitt and Secretary of the Board of Curators of Utrecht University, 27 January 1940, College van Curatoren, Utrecht Archives.

24 Board of Directors of the Municipal and Academic Hospital in Utrecht to Leendert Bouman, 10 April 1935, College van Curatoren, Utrecht Archives. In the first half of the twentieth century, the provision of healthcare in the Netherlands was stratified based on the patient’s socio-economic position. Third class patients, referred to as “the indigent” (“*onvermogenden*”) could not financially contribute to their own care and received only the most basic hospital facilities, the costs of which were typically covered by the municipality. Second class patients or “the less-wealthy” (“*minvermogenden*”) could make a modest financial contribution themselves, often with some form of insurance through local health insurance funds (*ziekenfondsen*), and received more hospital benefits (better room, better meals, more attention from medical and nursing staff). First class or “private” patients could privately cover their medical costs and received the most luxurious hospital facilities (private room, multi-course dinners, etc.). See Juch, *De medische specialisten in de Nederlandse gezondheidszorg*, 57-58.

25 Cornelis Lenshoek to the Board of Directors of the Municipal and Academic Hospital in Utrecht, 18 December 1939, College van Curatoren, Utrecht Archives.

26 Minutes of meeting between the Board of Directors of the Municipal and Academic Hospital in Utrecht, the Board of Curators of Utrecht University, and the Secretary-General of the Ministry of Education, Arts, and Sciences, undated, 1940, College van Curatoren, Utrecht Archives.

27 Instruction for the Neuro-Surgeon (*Instructie voor den Neuro-chirurg*), 29 February 1940, College van Curatoren, Utrecht Archives.

28 Willem Sillevis Smitt to the Board of Directors of the Municipal and Academic Hospital in Utrecht, 12 March 1940, College van Curatoren, Utrecht Archives.

29 For an analysis of the forced migration of European physicians and scientists working in the neuro disciplines as a result of World War II, see Frank Stahnisch, *A New Field in Mind: A History of Interdisciplinarity in the Early Brain Sciences* (Montréal: McGill-Queen’s University Press, 2020), 201–236.

30 Secretary-General of the Ministry of Education, Arts, and Science to Board of Curators of Utrecht University, 12 July 1940, College van Curatoren, Utrecht Archives.

31 Board of Curators of Utrecht University to the Secretary-General of the Ministry of Education, Arts, and Science, 8 July 1940, College van Curatoren, Utrecht Archives.

32 In contrast to the Board of Directors, the Board of Curators largely consisted of educated laymen, who were less informed about the state of development in the fields of neurology and neurosurgery, and were typically more invested in the educational matters than in clinical affairs. For the composition of the board of curators of Utrecht University in the middle of the twentieth century, see Hermann von der Dunk, Wybo Heere, and Adriaan Reinink, ed., *Tussen ivoren toren & grootbedrijf: de Utrechtse Universiteit, 1936-1986* (Maarssen: G. Schwartz, 1986), 536.

33 Minutes of meeting between the Board of Directors of the Municipal and Academic Hospital in Utrecht, the Board of Curators of Utrecht University, and the Secretary-General of the Ministry of Education, Arts, and Sciences, undated 1940, College van Curatoren, Utrecht Archives.

34 Board of Directors of the Municipal and Academic Hospital in Utrecht to Secretary-General of the Ministry of Education, Arts, and Sciences, 24 July 1940, College van Curatoren, Utrecht Archives.

35 For the concept of problem ownership, introduced by Joseph Gusfield to describe the way in which certain professions of manage to obtain legitimacy in dealing with specific social problems, see Joseph R. Gusfield, “Constructing the Ownership of Social Problems: Fun and Profit in the Welfare State,” *Social Problems* 36 (1989): 431–441.

36 For the “diagnostic identity” of Dutch neurologists in the late nineteenth and early twentieth century, see Lutters, “Division of Labor or a New Discipline,” 139–146.

37 For the separation of neurology and neurosurgery in the Netherlands, as evidenced by the installment of separate academic chairs in neurology and psychiatry during the first half of the twentieth century, see Hans de Waardt, “De konijntjes van Winkler. De scheiding van de leerstoelen neurologie en psychiatrie in Nederland,” *Gewina* 24 (2001): 157–168.

38 Brouwer, for instance, reflected on the dire state of neurology on multiple occasions. See Bernard Brouwer, “Over de toekomst der neurologie: voordracht bij de opening van het nieuwe Neurologische Instituut op 28 september 1929,” *Psychiatrische neurologische bladen* 34 (1930): 23-32; B. Brouwer, “Training of the Neurologist,” *Archives of Neurology and Psychiatry* 29 (1933): 624–632.

39 The jurisdiction of neurology was similarly challenged in America and other European countries. See Gavrus, “Men of Dreams and Men of Action,” 71–77; Stephen T. Casper, *The Neurologists: A History of a Medical Specialty in Modern Britain, C. 1789-2000* (Manchester: Manchester University Press, 2015).

40 Hendrik Stenvers, “Neurologie en neurochirurgie,” *Nederlands tijdschrift voor geneeskunde* 84 (1940): 4545–4547.

41 Ibid., 4545. Similar appeals to the fundamental differences in character between neurologists and neurosurgeons were made by American neurologists during the interwar period. See Gavrus, “Men of Dreams and Men of Action,” 75. Such comparisons drew on the age-old distinction between physicians as sophisticated gentlemen and surgeons as butchers. See Christopher Lawrence, “Medical Minds, Surgical Bodies: Corporeality and the Doctors,” in *Science Incarnate: Historical Embodiments of Natural Knowledge*, ed. Christopher Lawrence and Steven Shapin (Chicago: University of Chicago Press, 1998), 156–201.

42 Stenvers, “Neurologie en neurochirurgie,” 4546.

43 Ibid., 4546.

44 Ibid., 4547.

45 Arnold de Vet, “Neurologie en neurochirurgie,” *Nederlands tijdschrift voor geneeskunde* 84 (1940): 4844.

46 Ferdinand Verbeek, “Neurologie en neuro-chirurgie,” *Nederlands tijdschrift voor geneeskunde* 84 (1940): 5139.

47 Ibid.

48 Ibid.; Ferdinand Verbeek, “Reactie: Neurologie en neuro-chirurgie,” *Nederlands tijdschrift voor geneeskunde* 85 (1941): 294, emphasis in original.

49 Gavrus has identified a similar appeal to therapy by North American neurosurgeons as a means to challenge the authority of neurologists within a boarder social climate of “therapeutic headiness.” See Gavrus, “Men of Dreams and Men of Action,” 64. In the Netherlands, too, therapy was an important legitimizing principle for medical authority. In fact, Brouwer’s efforts to establish the first neurosurgery ward in the country had been primarily motivated by therapeutic concerns, as the internalization of neurosurgery held the promise of infusing neurology with therapeutic expertise. See Lutters, “Division of Labor or a New Discipline,” 146–150. For a an analysis of the relationship between therapeutic innovation and social change during the twentieth century, see Jeremy A. Greene, Flurin Condrau, Elizabeth Siegel Watkins, ed., *Therapeutic Revolutions: Pharmaceuticals and Social Change in the Twentieth Century* (Chicago: University of Chicago Press, 2016).

50 Verbeek, “Neurologie en neuro-chirurgie,” 5140.

51 Bernard Brouwer, *Kunst en kunde in de diagnostiek: rede bij de 310*^*de*^  *herdenking van den stichtingsdag der inrichting voor Hooger Onderwijs te Amsterdam, uitgesproken op 8 januari 1942* (Amsterdam: Stadsdrukkerij, 1942).

52 Ibid., 6.

53 Ibid., 4.

54 Ibid., 8. For the use of quantification strategies in Western medicine in the nineteenth and twentieth century, see J. Rosser Matthews, *Quantification and the Quest for Medical Certainty* (Princeton: Princeton University Press, 1995). For the broader endorsement of quantification methods in modern society, see Theodore M. Porter, *Trust in Numbers: The Pursuit of Objectivity in Science and Public Life* (Princeton: Princeton University Press, 1995).

55 “Kunst en kunde in de diagnostiek: stoffelijke zaken en voelende mensen,” *Het nationale dagblad*, 9 January 1942.

56 Peter Jan Knegtmans*, Een kwetsbaar centrum van de geest: de Universiteit van Amsterdam tussen 1935 en 1950* (Amsterdam: Amsterdam University Press, 1998), 149.

57 Bernard Brouwer, *Jaarverslag van de lotgevallen der Universiteit van Amsterdam gedurende den cursus 1941-1942* (Amsterdam: Stadsdrukkerij, 1942), 9.

58 Ibid.

59 Ferdinand Verbeek, “Overzicht: 10-jaren neuro-chirurgische studiekring,” 2, 9 November 1946, Box 1, Notulen 1936-1949/Correspondentie 1936-1990 Studieclub voor Neuro-chirurgie, Archives NVvN.

60 Gavrus has described a similar “exclusionist ethos” amongst the first generation of American neurosurgeons, which enabled them to cultivate specific professional and moral qualities at the meetings of the Society for Neurological Surgeons. See Gavrus, “Skill, Judgement and Conduct for the First Generation of Neurosurgeons,” 366. Even though in the Netherlands general surgeons started to abandon the practice of brain surgery after the establishment of Oljenick in 1929, this was a gradual process and some surgeons strongly resisted (*op. cit.*, note 15). Moreover, surgery of the spinal cord and peripheral nervous system remained part of the general surgeons’ jurisdiction throughout the first half of the twentieth century. See for instance, Bernard Brouwer, *Over de ontwikkeling der neuro-chirurgie* (Amsterdam: J.B. Wolters, 1947), 15.

61 In the second half of the 1920s, Brouwer had already feared that he would not have enough work for “his neurosurgeon.” See Brouwer, *Over de ontwikkeling der neuro-chirurgie*, 11. Similarly in 1937, in a letter to the Specialist Registration Committee, Brouwer stated that a total of four neurosurgeons would be sufficient to cover the demand for neurosurgery for the following ten years. See Bernard Brouwer to the secretary of the Specialist Registration Committee, 25 March 1937, Box 1, Notulen 1936-1949/Correspondentie 1936-1990 Studieclub voor Neuro-chirurgie, Archives NVvN.

62 For the exchange of tacit knowledge among American neurosurgeons at the first meetings of the Society of Neurological Surgeons, see Gavrus, “Skill, Judgement and Conduct for the First Generation of Neurosurgeons,” 374–375.

63 Historian Ludmilla Jordanova has described the way in which physicians used visual display as tangible evidence of their professional credibility. See Ludmilla Jordanova, “Medicine and Genres of Display,” in *Visual Display: Culture Beyond Appearances*, ed. Lynne Cooke and Peter Wollen (Seattle: New Press, 1995), 203.

64 Address by Arnold de Vet at the occasion of the 35th anniversary of the Study Club for Neurosurgery, 14 April 1972, Box 1, Notulen 1936-1949/Correspondentie 1936-1990 Studieclub voor Neuro-chirurgie, Archives NVvN. For a similar “mixture of the professional and the entertaining” at the meetings of the American Neurological Association during the interwar period, see Gavrus, “Men of Dreams and Men of Action,” 82-83. Medical historian Thomas Schlich has also described a “fraternity culture” of friendship and conviviality amongst Swiss surgeons dedicated to bone fracture treatment in the postwar period. See Thomas Schlich, *Surgery, Science and Industry: A Revolution in Fracture Care, 1950s–1990s* (New York: Palgrave MacMillan, 2002), 35–41.

65 Specialist Registration Committee to Bernard Brouwer, 15 March 1937, Box 1, Notulen 1936-1949/Correspondentie 1936-1990 Studieclub voor Neuro-chirurgie, Archives NVvN.

66 See for instance, Minutes of the Study Club for Neuro-Surgery, 23 October 1948, Box 1, Notulen 1936 -1949/Correspondentie 1936-1990 Studieclub voor Neuro-chirurgie, Archives NVvN.

67 The question whether nervous system surgery primarily belonged to the domain of surgery or neurology had been raised before in the Netherlands. In the first decades of the twentieth century, the Dutch neurologist Louis Muskens (1872-1937) had widely advocated that nervous system surgery was to be performed by neurologists rather than general surgeons, a notion that had been rejected by neurologists and surgeons alike. See Lutters, “Division of Labor or a New Discipline.”

68 Bernard Brouwer to the secretary of the Specialist Registration Committee, 25 March 1937, Box I, Notulen 1936-1949/Correspondentie 1936-1990 Studieclub voor Neuro-chirurgie, Archives NVvN.

69 Ferdinand Verbeek to Arnold de Vet, 20 August 1937, Box 1, Notulen 1936-1949/Correspondentie 1936-1990 Studieclub voor Neuro-chirurgie, Archives NVvN.

70 See for instance, Willem Sillevis Smitt to Bernard Brouwer, 28 April 1942, Box I, Notulen 1936-1949/Correspondentie 1936-1990, Archives of the Study Club for Neuro-Surgery. Sillevis Smitt’s insistence on Verbiest’s membership showed that individual members of the Study Club were not simply driven by common overarching professional interests, but also by personal and local interests, which occasionally led to disagreements amongst neurologists and neurosurgeons themselves.

71 See for instance, Minutes of the Study Club for Neuro-Surgery, 23 October 1948, Box 1, Notulen 1936-1949/Correspondentie 1936-1990 Studieclub voor Neuro-chirurgie, Archives NVvN.

72 Address by Arnold de Vet, 14 April 1972, Box II, Notulen 1950-1989 Studieclub voor Neuro-chirurgie, Archives NVvN.

73 “Henk Verbiest, MD interviewed by Roy C. Selby, MD,” American Association of Neurological Surgeons, filmed in 1992, video, 43:17, https://www.youtube.com/watch?v=hA_0Zi9MuqA.

74 Van der Maaden’s cartoons provide a rich and previously unexplored source for understanding the lay perception of Dutch psychiatric, neurological, and neurosurgical patient care in the middle of twentieth century. For an analysis of public attitudes towards medicine in the late nineteenth and first half of the twentieth century as revealed by popular images such as cartoons, see Bert Hansen, *Picturing Medical Progress from Pasteur to Polio: A History of Mass Media Images and Popular Attitudes in America* (New Brunswick, NJ: Rutgers University Press, 2009).

75 Knegtmans, *Een kwetsbaar centrum van de geest*, 242–254.

76 Verbeek, “Overzicht: 10-jaren neuro-chirurgische studiekring,” 3.

77 In 1941, the German occupiers issued a decree that required every Dutch citizen to join a Health Maintenance Organization (*ziekenfonds*). This decree was perpetuated by the Dutch government after the war, and gave people who had previously been unable to afford it Health Maintenance Organization membership access to healthcare, resulting in a dramatic increase in the demand for healthcare. See Hein A.M. Klemann, *Nederland 1938-1948: Economie en samenleving in jaren van oorlog en bezetting* (Amserdam: Boom, 2002), 409–410.

78 Verbeek, “Overzicht: 10-jaren neuro-chirurgische studiekring,” 2.

79 For a historical analysis of the characteristic depiction of surgeons as uncivilized and bulky craftsmen or butchers, in contrast to the thoughtful and slender depiction of physicians, see Lawrence, “Medical Minds, Surgical Bodies,” 156–201.

80 Board of Curators of the University of Groningen to the Ministry of Education, Arts, and Sciences, 7 February 1947, Inventory 1245, Directie-archief Algemeen Provinciaal Stads en Academisch Ziekenhuis Groningen 1903-1989, Archives of the University Medical Center Groningen, Groningen, the Netherlands (hereafter cited as Directie-archief, Groningen).

81 Board of Curators of the University of Groningen to the Ministry of Education, Arts, and Sciences, 15 February 1946, catalogue reference 2.14.17, inventory 145, Inventaris van het archief van het Ministerie van Onderwijs, Kunsten en Wetenschappen: Persoonsdossiers Rijkspersoneel, ca. 1870-ca. 1974, National Archives of the Netherlands, The Hague, The Netherlands (hereafter cited as Ministerie van Onderwijs, Kunsten en Wetenschappen, National Archives).

82 Ministry of Education, Arts, and Sciences to Board of Curators of the University of Groningen, 27 April 1946, Ministerie van Onderwijs, Kunsten en Wetenschappen, National Archives.

83 Ferdinand Verbeek to Bernard Brouwer, 10 September 1947, Box 1, Notulen 1936-1949/Correspondentie 1936-1990 Studieclub voor Neuro-chirurgie, Archives NVvN, underlined in the original.

84 Minutes of the Study Club for Neuro-Surgery, 8 November 1947, Box 1, Notulen 1936-1949/Correspondentie 1936-1990 Studieclub voor Neuro-chirurgie, Archives NVvN.

85 Secretary of Faculty of Medicine of Utrecht University to the Board of Curators of Utrecht University, 18 November 1947, catalogue reference 59, inventory 901, College van Curatoren van de Rijksuniversiteit Utrecht, Utrecht Archives (hereafter cited as 59/901 College van Curatoren van de Rijksuniversiteit Utrecht, Utrecht Archives).

86 Secretary of Faculty of Medicine of Utrecht University to Board of Curators of Utrecht University, 27 January 1948, 59/901 College van Curatoren van de Rijksuniversiteit Utrecht, Utrecht Archives.

87 Memo of the Department of Higher Education of the Ministry of Education, Arts and Sciences, 8 September 1948, Ministerie van Onderwijs, Kunsten en Wetenschappen, National Archives.

88 Dutch neurosurgeons to Bernard Brouwer, October 1948, Box 1, Notulen 1936-1949/Correspondentie 1936-1990 Studieclub voor Neuro-chirurgie, Archives NVvN.

89 Joseph Prick, “Memorandum,” October 1948, Box 1, Notulen 1936-1949/Correspondentie 1936-1990 Studieclub voor Neuro-chirurgie, Archives NVvN. Myelography was an invasive diagnostic procedure in which a contrast agent was injected in the spinal cerebrospinal fluid compartment. With the technique, lesions within or around the spinal cord could be identified on the radiogram. For an overview of the invention and subsequent refinement of the procedure, see Bart Lutters, Rob Groen, and Peter Koehler, “Myelography and the 20th Century Localization of Spinal Cord Lesions,” *European Neurology* 83 (2020): 447–452.

90 Arie Biemond, *Enige beschouwingen over erfelijke organische zenuwziekten* (Haarlem: De Erven F. Bohn, 1947), 6–7.

91 Arie Biemond, “Memorandum,” October 1948, Box 1, Notulen 1936-1949/Correspondentie 1936-1990 Studieclub voor Neuro-chirurgie, Archives NVvN.

92 Minutes of the Study Club for Neuro-Surgery, 23 October 1948, Box 1, Notulen 1936-1949/Correspondentie 1936-1990 Studieclub voor Neuro-chirurgie, Archives NVvN.

93 Ministry of Education, Arts and Sciences to Board of Curators of the University of Groningen, 30 November 1948, Ministerie van Onderwijs, Kunsten en Wetenschappen, National Archives.

94 Arie van der Zwan, *De neuro-chirurgie als zelfstandig specialisme* (Groningen: J. B. Wolters, 1949).

95 Henk Verbiest, *De ontwikkeling in de neurochirurgische gedragingen* (Amsterdam: Scheltema & Holkema’s Boekhandel en Uitgeversmaatschappij, 1949). The first generation of American neurosurgeons fashioned a similar “highly cultured” image, with Cushing as the “epitome of the highly cultured, elite doctor with scholarly interests, of famous equanimity.” See Thomas Schlich, “‘The Days of Brilliancy are Past’: Skill, Styles and the Changing Rules of Surgical Performance, ca. 1820–1920,” *Medical History* 59 (2015): 399.

96 Verbiest, *De ontwikkeling in de neurochirurgische gedragingen*, 18.

97 For an analysis of the holistic concepts employed by neurologists during the first half of the twentieth century, see Anne Harrington, “Kurt Goldstein’s Neurology of Healing and Wholeness: A Weimar Story,” in *Greater Than the Parts: Holism in Biomedicine, 1920–1950*, ed. Christopher Lawrence and George Weisz (Oxford: Oxford University Press, 1998), 25–45. Such holistic concepts also permeated the field of nervous system surgery. See for instance, Katja Guenther, *Localization and Its Discontents: A Genealogy of Psychoanalysis and the Neuro Disciplines* (Chicago: University of Chicago Press, 2015), 96–125. Thomas Schlich has described how, during the late nineteenth and early twentieth centuries, laboratory science became the new epistemic foundation of surgery and neurosurgery. See Thomas Schlich, “Physiological Surgery: Laboratory Science as the Epistemic Basis of Modern Surgery and Neurosurgery,” in *The History of the Brain and Mind Sciences*, 48–76. In the Netherlands, the adoption of physiological surgery was somewhat delayed. See Lutters, “Division of Labor of a New Discipline,” 132–133. In fact, the Dutch neurosurgeons lamented on multiple occasions that their clinical obligations left no time for scientific research, which they blamed on their neurological supervisors who had allegedly left the neurosurgery wards hopelessly understaffed. See for instance, Dutch neurosurgeons to Bernard Brouwer, October 1948, Box 1, Notulen 1936-1949/Correspondentie 1936-1990 Studieclub voor Neuro-chirurgie, Archives NVvN.

98 Joseph Prick, “Het onderwijs in de psychiatrie en neurologie en de vorming tot zenuwarts in het perspectief van verleden en toekomst,” *R.K. artsenblad* 28 (1949): 41. Such parent-child metaphors were also employed by other Dutch neurologists to claim their legitimate share in neurosurgical patients care. In his inaugural address, for instance, Biemond argued that the Dutch neurosurgeons’ quest for independence could turn the specialty into neurology’s “problem child.” See Biemond, *Enige beschouwingen over erfelijke organische zenuwziekten*, 6. American neurologists also evoked parent-child metaphors in jurisdictional conflicts with their neurosurgical colleagues. See Gavrus, “Men of Dreams and Men of Action,” 69.

99 Prick, “Het onderwijs in de psychiatrie en neurologie en de vorming tot zenuwarts in het perspectief van verleden en toekomst,” 41.

100 Cornelis Lenshoek, “Memorial speech Professor Dr. B. Brouwer,” delivered at the meeting of the Study Club, 12 November 1949, Box 1, Notulen 1936-1949/Correspondentie 1936-1990 Studieclub voor Neuro-chirurgie, Archives NVvN.

101 The Dutch neurosurgeons to Director of the Municipal hospital at the Coolsingel in Rotterdam, 22 August 1952, Box 1, Notulen 1936-1949/Correspondentie 1936-1990 Studieclub voor Neuro-chirurgie, Archives NVvN.

102 Director of the Municipal Hospital at the Coolsingel in Rotterdam to the Dutch neurosurgeons, 26 November 1952, Box 1, Notulen 1936-1949/Correspondentie 1936-1990 Studieclub voor Neuro-chirurgie, Archives NVvN.

103 Dutch neurosurgeons to the neurological members of the Study Club for Neuro-Surgery, November 1952, Box 1, Notulen 1936-1949/Correspondentie 1936-1990 Studieclub voor Neuro-chirurgie, Archives NVvN. The possessive name “the Netherlands Society of Neurosurgeons” aptly reflected the neurosurgeons’ commitment to break with the neurological hegemony that had characterized the negotiation of neurosurgical patient care in the Netherlands for over two decades.

104 Cornelis Lenshoek, *Van antithese naar synthese in de neurochirurgie* (Groningen: J. B. Wolters, 1956). Similar to Van der Zwan’s position as lector, the installment of neurosurgery’s first professorship primarily resulted from the ongoing competition between the various academic hospitals in the country, as the promise of a professorship had allowed the academic hospital in Groningen to attract an experienced neurosurgeon upon Van der Zwan’s sudden resignation.

105 Ibid., 16–17.

